# Myriad untold stories unfolding daily: South Africa’s pursuit of quality primary health care

**DOI:** 10.4102/safp.v66i1.5867

**Published:** 2024-01-31

**Authors:** Klaus B. von Pressentin, Ramprakash Kaswa, Shane Murphy, Arun Nair, Indiran Govender

**Affiliations:** 1 Department of Family, Community and Emergency Care, Faculty of Health Sciences, University of Cape Town, Cape Town, South Africa; 2 Department of Family Medicine and Rural Health, Walter Sisulu University, Mthatha, South Africa; 3 Mthatha General Hospital, Mthatha, South Africa; 4 Outpatient Department, Clinical Services, Abbey House Medical Centre, Navan, Ireland; 5 Department of Family Medicine, University of the Free State, Kimberley, South Africa; 6 Robert Mangaliso Sobukwe Hospital, Northern Cape Department of Health, Kimberley, South Africa; 7 Department of Family Medicine and Primary Health Care, School of Medicine, Sefako Makgatho Health Sciences University, Pretoria, South Africa

In the dynamic healthcare landscape, where political ideals and invested personal interests intersect with financial realities, primary care teams, particularly family physicians, are at the forefront of quality standards implementation. The urgency intensifies as we prepare for the National Health Insurance (NHI) and Universal Health Coverage (UHC). Recent discussions at global platforms like the United Nations General Assembly and the World Health Summit have underscored the pressing need for high-quality primary health care (PHC) to guarantee UHC.^1^ The question resonates: What does quality genuinely mean, especially in the context of South African communities? This conversation accentuates the need for professional and ethical frameworks within quality healthcare.

As our country prepares for UHC through NHI, the urgency to address the ethical and service delivery challenges within our healthcare system intensifies. Current policies and fiscal constraints have reshaped the healthcare landscape, emphasising the need for robust healthcare leadership and governance. The 2019 Health Market Inquiry into the Private Healthcare Sector sheds light on rising costs, overutilisation and the lack of demonstrable associated improvements in health outcomes.^2^ Both public and private sectors need help delivering quality healthcare in the context of unequal resource distribution, management and leadership crises, increasing disease burdens and slow progress in health system reforms.^3^ Also, the current national policy on quality in healthcare needs to be updated (published in 2001, revised in 2007).^4^ Encouragingly, the Office of Health Standards Compliance has defined the quality standards for South African health facilities to support the journey to UHC; however, it is essential to reflect on how implementing these standards is translated into an enhanced experience for healthcare users. It is crucial to understand the lived reality at the PHC level and acknowledge the pivotal role family physicians, general practitioners and other primary care providers play in leading quality improvement initiatives.

Empowering the primary care team necessitates dynamic leadership and governance to create safe spaces for open dialogue and nurturing a culture of continuous quality improvement. Leadership and clinical governance are integral to the postgraduate training of specialist family physicians, aligning with the South African national unit standards.^5^ The quest for a ‘shared vocabulary’ in uncertain times was reiterated by Dr Anna Stavdal, the World Organization of Family Doctors (WONCA) President, during her plenary address at the 2023 WONCA World Conference.^6^ She also mentioned the ongoing WONCA ‘Global Core Values Project’ to affirm our core values as a discipline from a global perspective, as:

[*S*]hedding light on the core values that WONCA stands for can help us define our shared identity and thus increase our visibility. That, in turn, broadens our opportunities to exert influence.^7^

Central to this discussion is the incorporation of virtue-based ethics and values-driven leadership.^8^ Primary care clinicians, particularly family physicians, must embody empathy, integrity and compassion. These virtues guide ethical decision-making, fostering trust and understanding between clinicians, patients and communities. By cultivating these virtues, clinicians can navigate the complex terrain of ethical dilemmas, ensuring that the delivery of care aligns with both clinical expertise and moral integrity. Yet, the establishment of a culture of quality is full of challenges, particularly in contexts where diverse values intersect. The clash of values often arises when cultural beliefs, patient preferences, limited resources and ethical principles diverge. Addressing this clash necessitates open dialogue, cultural competence and humility and a profound respect for diverse perspectives.

We must amplify the ‘behind the scenes’ roles of family physicians and their primary care teams. Equally vital is the patient voice – a powerful force that can illuminate aspects that might go unnoticed otherwise. Patients and community members must feel empowered to voice their concerns, ensuring that quality is not just a set of standards but an experience lived and felt by each individual seeking healthcare. In the journey towards UHC, the definition of quality may be in the eye of the beholder. Still, the commitment to achieving it must be unanimous. The lived reality at the grassroots level is that there is a myriad of untold stories unfolding daily, where primary care teams embody this commitment that ensures every patient receives the best care possible every time they walk through our doors. A recent series of short reports documented some of these experiences^9^; however, a simple descriptive analysis of published research in this journal highlighted the need for more research on rural settings, children and adolescents, observational analytical and experimental research and more outputs from interdisciplinary teams.^10^ More advocacy efforts are needed using all available platforms and channels, reiterated by Dr Mark Heywood at the 25th Annual National Family Practitioners Congress in 2023.

As primary care clinicians, educators and researchers, our responsibility is clear: to navigate ethical challenges, embrace complexity and amplify the voices of our patients. By fostering a culture of accountability and continuous improvement of person-centred care, we can redefine ‘quality’ healthcare in Southern Africa. Let the 26th Annual National Family Practitioners Congress from 06 to 08 September 2024 in Cape Town be the crucible for innovative solutions, shared wisdom and transformative ideas. Let us utilise this opportunity to create a path for a future where quality, ethical practices and community advocacy are not just buzzwords but an intrinsic part of our healthcare system.
